# Supplementary material for: Observation of formation and local structures of metal-organic layers via complementary electron microscopy techniques
Source: Nat Commun. 2022 Sep 3;13:5197. doi: 10.1038/s41467-022-32330-z (PMC9440887; doi:10.1038/s41467-022-32330-z)
Supplement: Supplementary file 1 — Supplementary Information [file 41467_2022_32330_MOESM1_ESM.pdf]

Supplementary Information

for

Observation of Formation and Local Structures of Metal-Organic Layers via Complementary Electron Microscopy Techniques

Xinxing Peng[1,#], Philipp M. Pelz[1,2,#], Qiubo Zhang[3,#], Peican Chen[4], Lingyun Cao[4], Yaqian Zhang[1,2], Hong-Gang Liao[4,*], Haimei Zheng[2,3], Cheng Wang[4], Shi-Gang Sun[4], Mary C. Scott[1,2,*]

Affiliations:

[1]National Center for Electron Microscopy, Molecular Foundry, Lawrence Berkeley National Laboratory, Berkeley, California 94720, United States

[2]Department of Materials Science and Engineering, University of California, Berkeley, California 94720, United States

[3]Materials Science Division, Lawrence Berkeley National Laboratory, Berkeley, California 94720, United States

[4]State Key Lab of Physical Chemistry of Solid Surfaces, Collaborative Innovation Center of Chemistry for Energy Materials, College of Chemistry and Chemical Engineering, Xiamen University, Xiamen, 361005, P. R. China

*To whom correspondence should be addressed; E-mail: mary.scott@berkeley.edu; hgliao@xmu.edu.cn

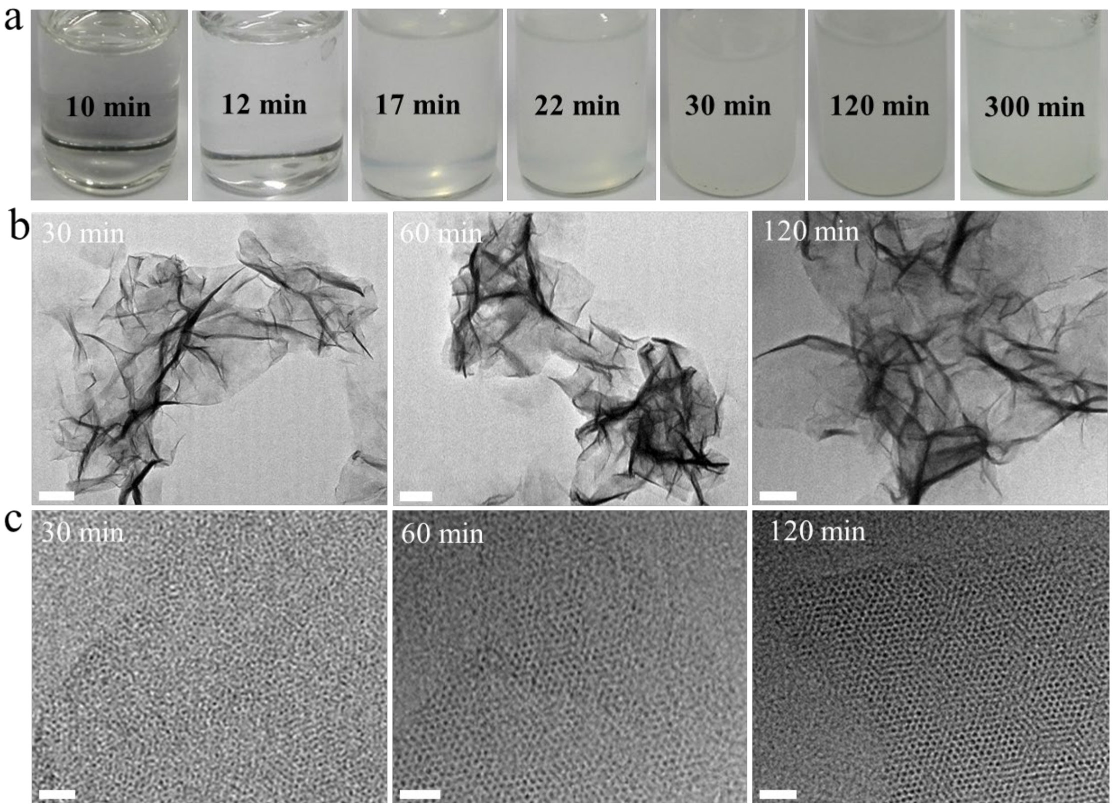

Supplementary Fig. 1 Hf-MOLs nanosheets ex-situ growth and intermediate product characterization. a Picture of the growth solution after baking at 120 °C for 10 min, 12 min, 17 min, 22 min, 30 min, 120 min and 300 min. **b** Low magnification and **c** high magnification TEM images of the Hf-MOLs after baking for 30 min, 60 min and 120 min. The dose rates for low magnification "30 min", "60 min", and "120 min" are 0.39, 0.39 and 0.79 $e^{-}\cdot Å^{-2}\cdot s^{-1}$, respectively. The dose rate for all high-magnification images in c is 285 $e^{-}\cdot Å^{-2}\cdot s^{-1}$. Scale bars: b 200 nm; c 10 nm.

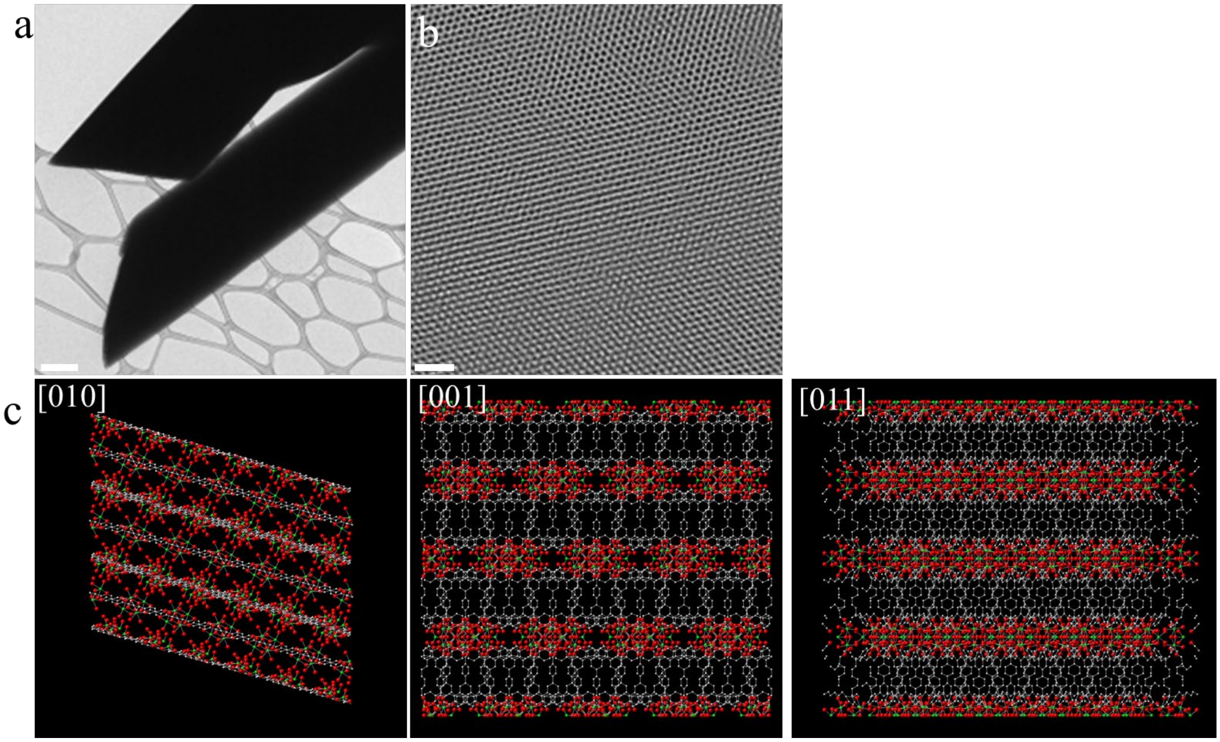

Supplementary Fig. 2 Structure characterization and atomic models of 3D Hf-MOFs. a Low- and **b** high-magnification TEM images of 3D Hf-MOFs. **c** Atomic models of 3D-MOFs viewing along [010], [001] and [011] directions. Green, red, white and pink spheres represent Hf, O, C, and H atoms, respectively. Scale bars: a 1 μm, b 10 nm.

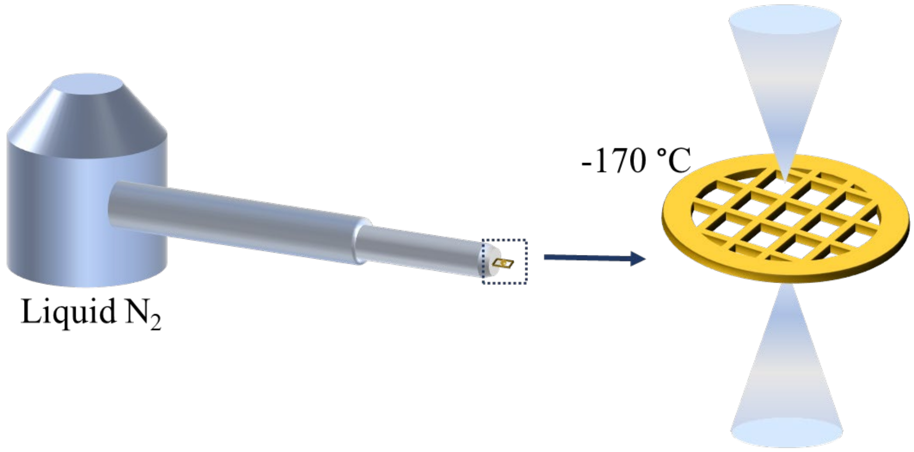

Supplementary Fig. 3 Experimental setup for sample characterization using cryo-holder. The sample was loaded into the microscope at room temperature. Then, the liquid nitrogen was filled into the cryo-holder to cool the sample. After more than 1.5 h, the temperature of the specimen will drop to -170 °C. We start to collect data after the cryo-holder has been stabilized.

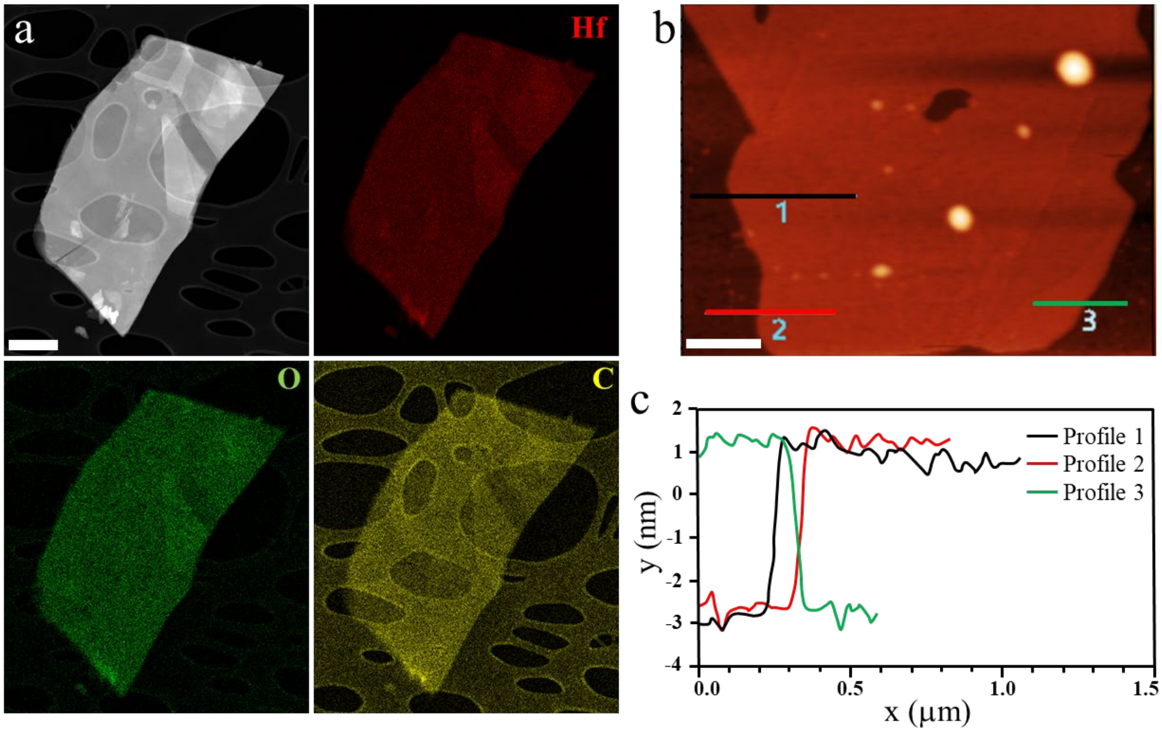

Supplementary Fig. 4 STEM-EDS mapping and thickness measurement of a multilayer region. a Representative HAADF-STEM image and the corresponding elemental maps of Hf, C and O. **b** Tapping-mode AFM topography of Hf-MOLs for a multilayer region. **c** Height profile along the black line (profile 1), red line (profile 2) and green line (profile 3). Scale bar: a, b 500 nm.

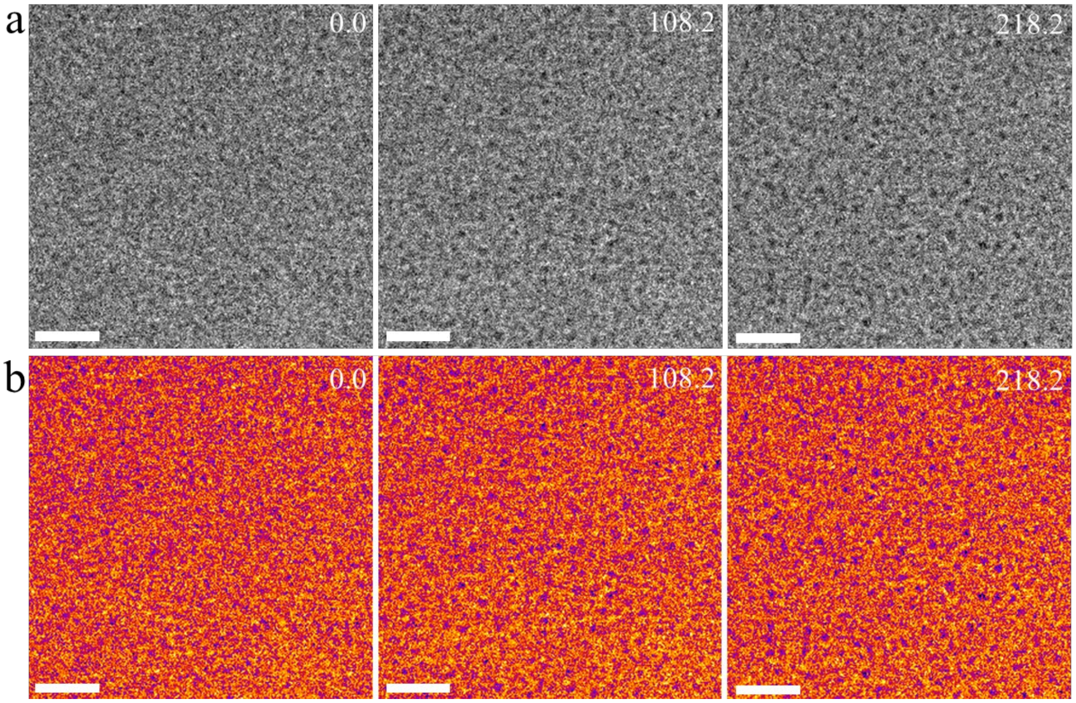

Supplementary Fig. 5 In-situ formation of Hf-clusters. a Snapshots from move S1 show the Hf-clusters' formation. **b** Their contrast was processed to be more directly interpretable by coloring. Scale bar 20 nm.

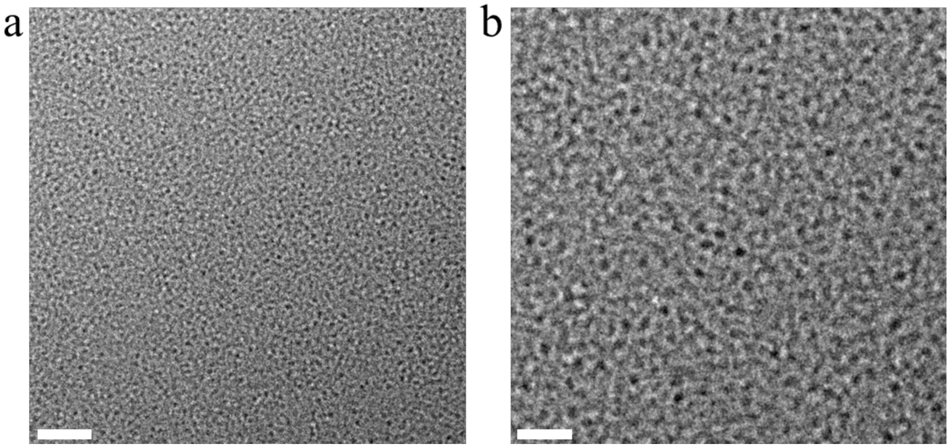

Supplementary Fig. 6 Characterization of Hf-clusters by HRTEM. a-b HRTEM images of the early-stage product after baking at 120 °C for 20 min. Clusters are clearly observed from the HRTEM images. Scale bars: a 20 nm; b 10 nm.

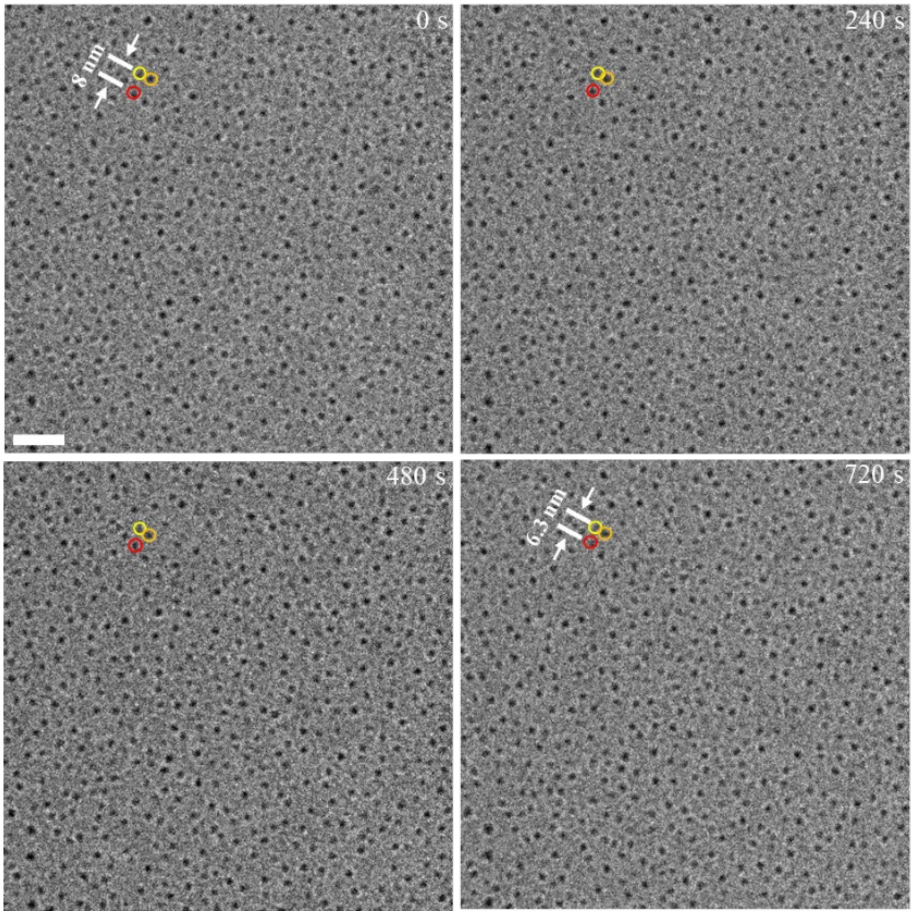

Supplementary Fig. 7 Sequential TEM images showing the slow-motion of clusters. Three clusters are selected to show clusters move randomly. The distance between the cluster in the red circle and the cluster in the yellow circle changes from 8 nm to 6.3 nm. Scale bar: 20 nm.

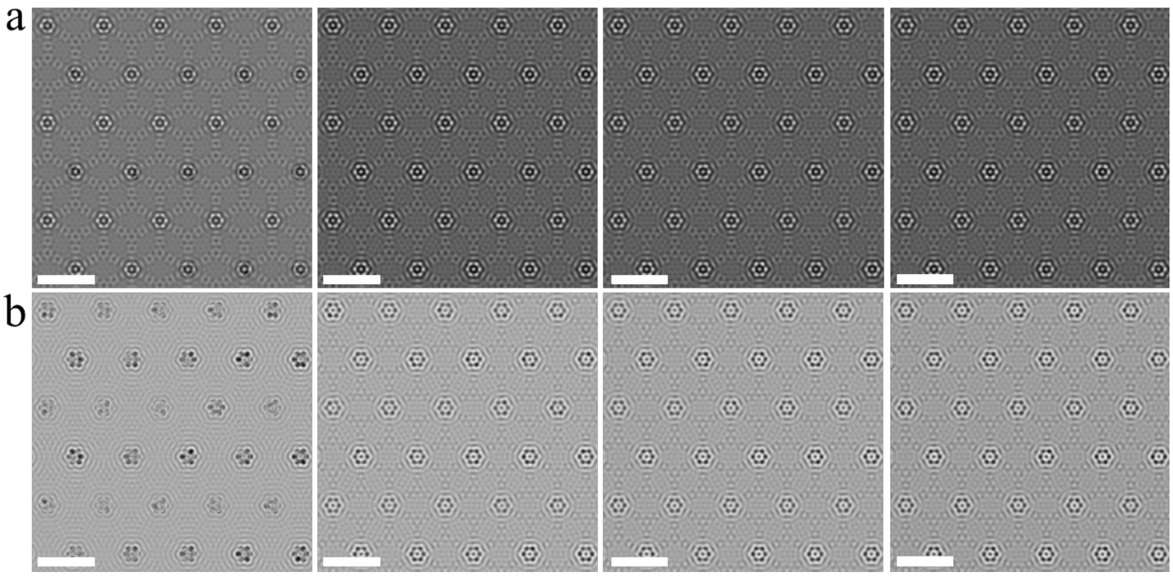

Supplementary Fig. 8 Simulated TEM image of Hf-MOLs. a Simulated TEM images of theoretical Hf-MOLs model at defocus of -1 nm, -3 nm, -5 nm and -7 nm (from left to right). **b** Simulated TEM images of theoretical Hf-MOLs model at defocus of 0 nm, 2 nm, 4 nm and 6 nm (left to right). Scale bars: a, b 2 nm.

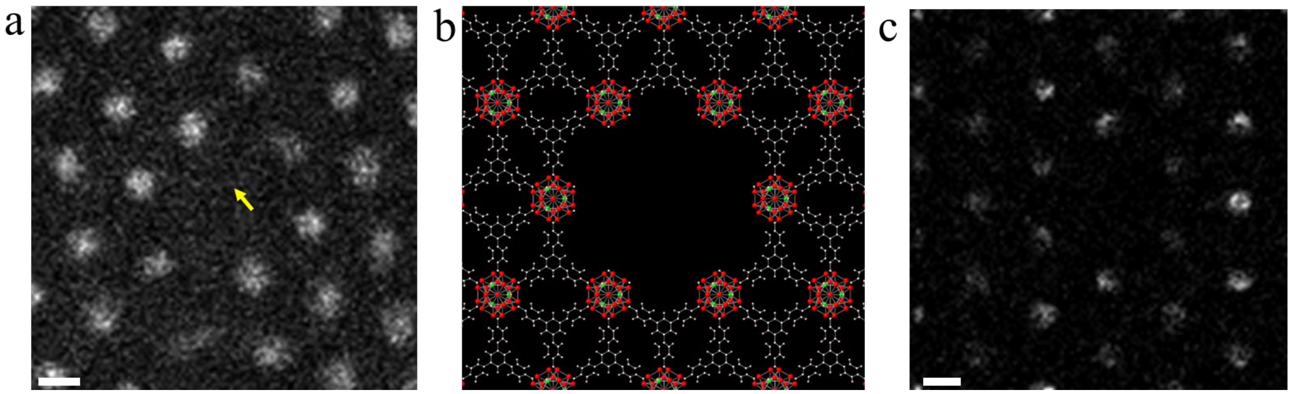

Supplementary Fig. 9 HAADF-STEM analysis of the region with a missing cluster. a HAADF-STEM image of MOLs shows the missing cluster (indicated by a yellow arrow). **b** Structural model of the point defect region within Hf-MOLs. Green, red, white and pink spheres represent Hf, O, C, and H atoms. **c** Simulated HAADF-STEM image of the missing cluster region. Scale bar: a, c 1 nm.

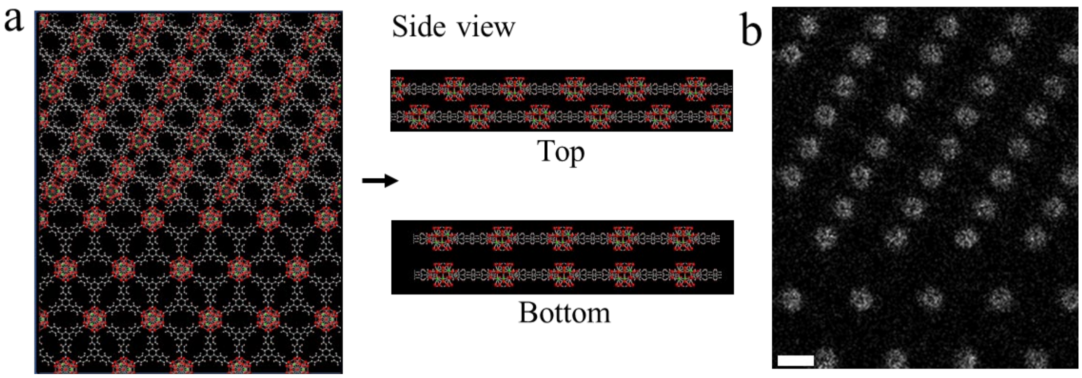

Supplementary Fig. 10 Structural models show the stacking between different layers. a Structural model shows the interface formed by different layer stacking behaviors. Green, red, white, and pink spheres represent Hf, O, C, and H atoms. **b** Simulated HAADF-STEM image of the stacking in **a**. Scale bar for b is 1nm.

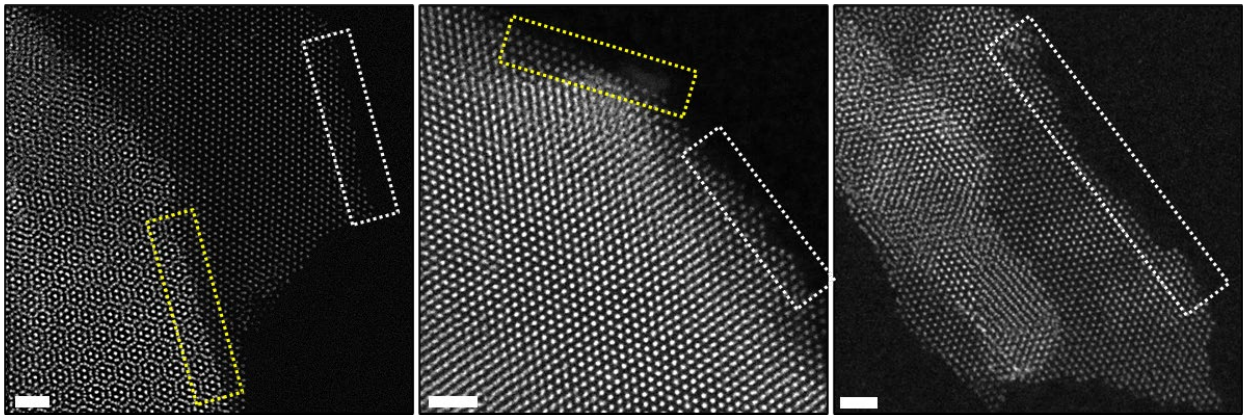

Supplementary Fig. 11 Revealing the structure of surface termination. HAADF-STEM image of MOLs shows the flat surface termination (indicated by the yellow square) and loop surface termination (indicated by the white square). Scale bars: 10 nm.

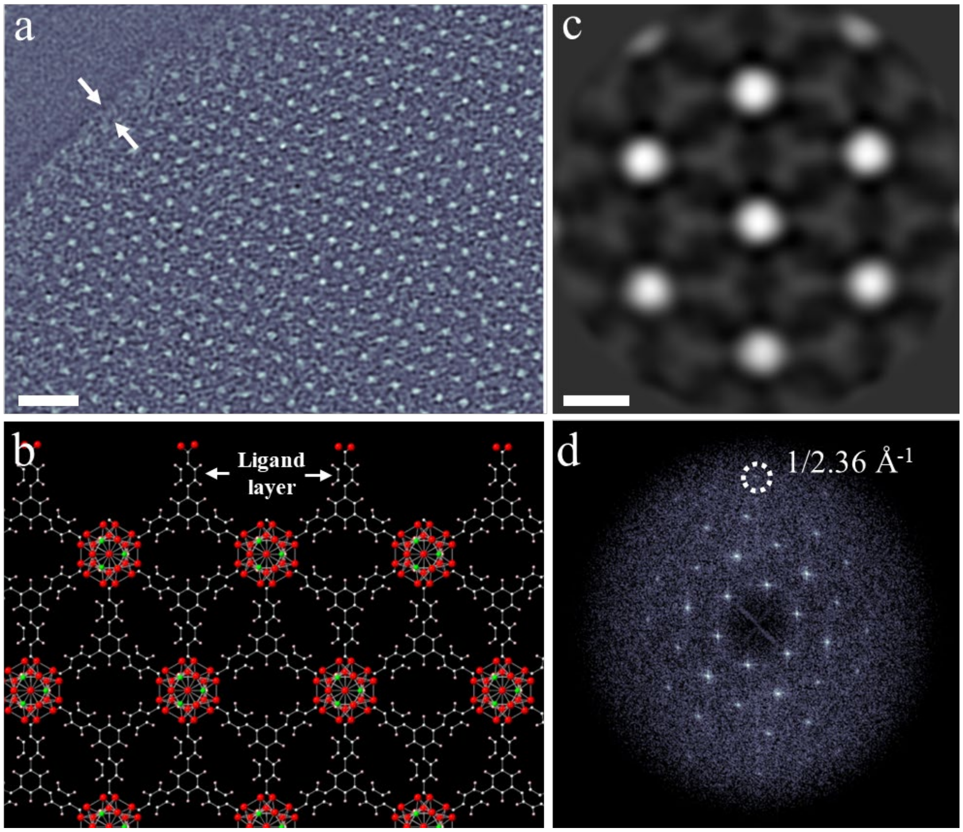

Supplementary Fig. 12 Electron ptychographic reconstruction of Hf-MOLs. a Ptychographic reconstructed phase image of the surface of the MOLs. The white arrow shows the nanoscale surface ligand layer. **b** Structural model of the Hf-MOL surface with ligand layer. Green, red, white and pink represent Hf, O, C and H atoms, respectively. **c** 2D class average of the reconstructed phase image. The image shows Hf-clusters and BTB ligands. **d** Intensity of Fourier transform of the reconstructed phase image, in which the dashed white circle represents information transfer to 1/2.36 $Å^{-1}$. Scale bars: a 5 nm; c 1 nm.

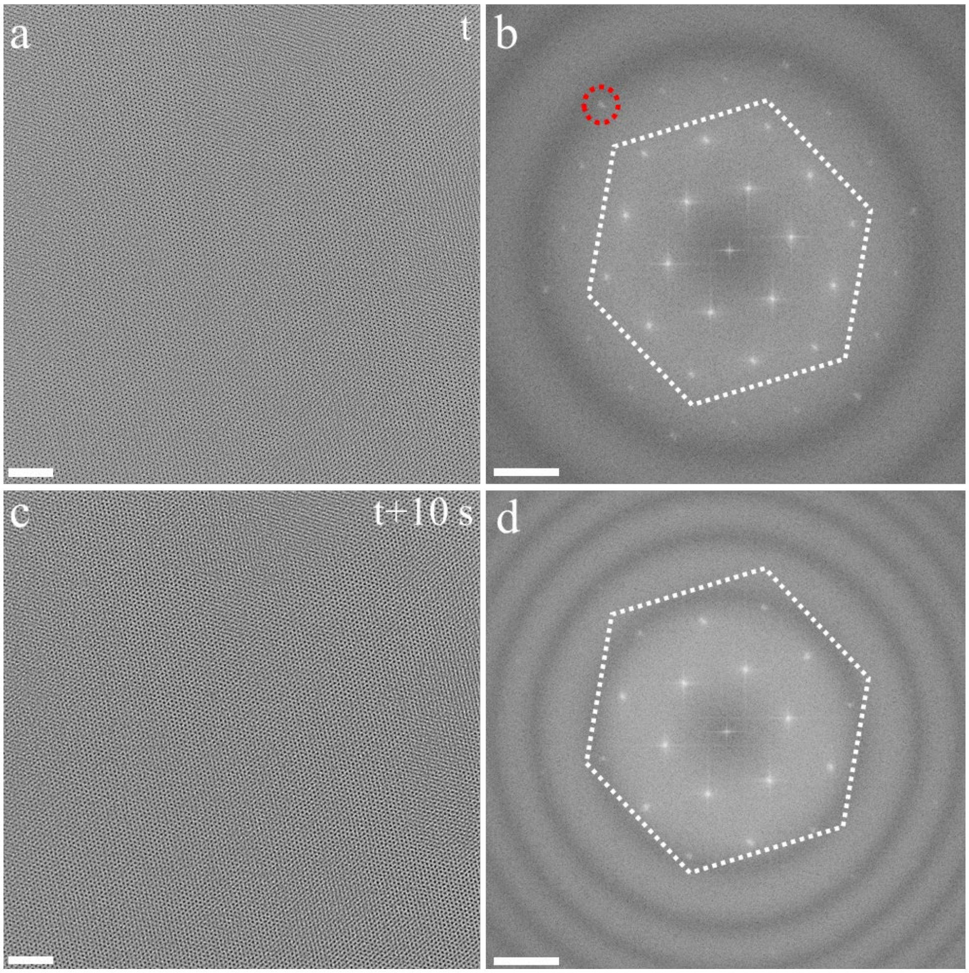

Supplementary Fig. 13 Stability test of Hf-MOLs under electron-beam irradiation at low temperature in TEM mode. a HRTEM image of MOLs acquired at the electron dose rate of 3200 $e^{-}\cdot Å^{-2}\cdot s^{-1}$. **b** FFT of the whole TEM image in a. **c** HRTEM image of MOLs acquired at a dose of 3200 $e^{-}\cdot Å^{-2}\cdot s^{-1}$ after being exposed to the electron beam for 10 s. **d** FFT of the whole TEM image in c. Scale bar: a, c 20 nm; b, d 0.5 1/nm.

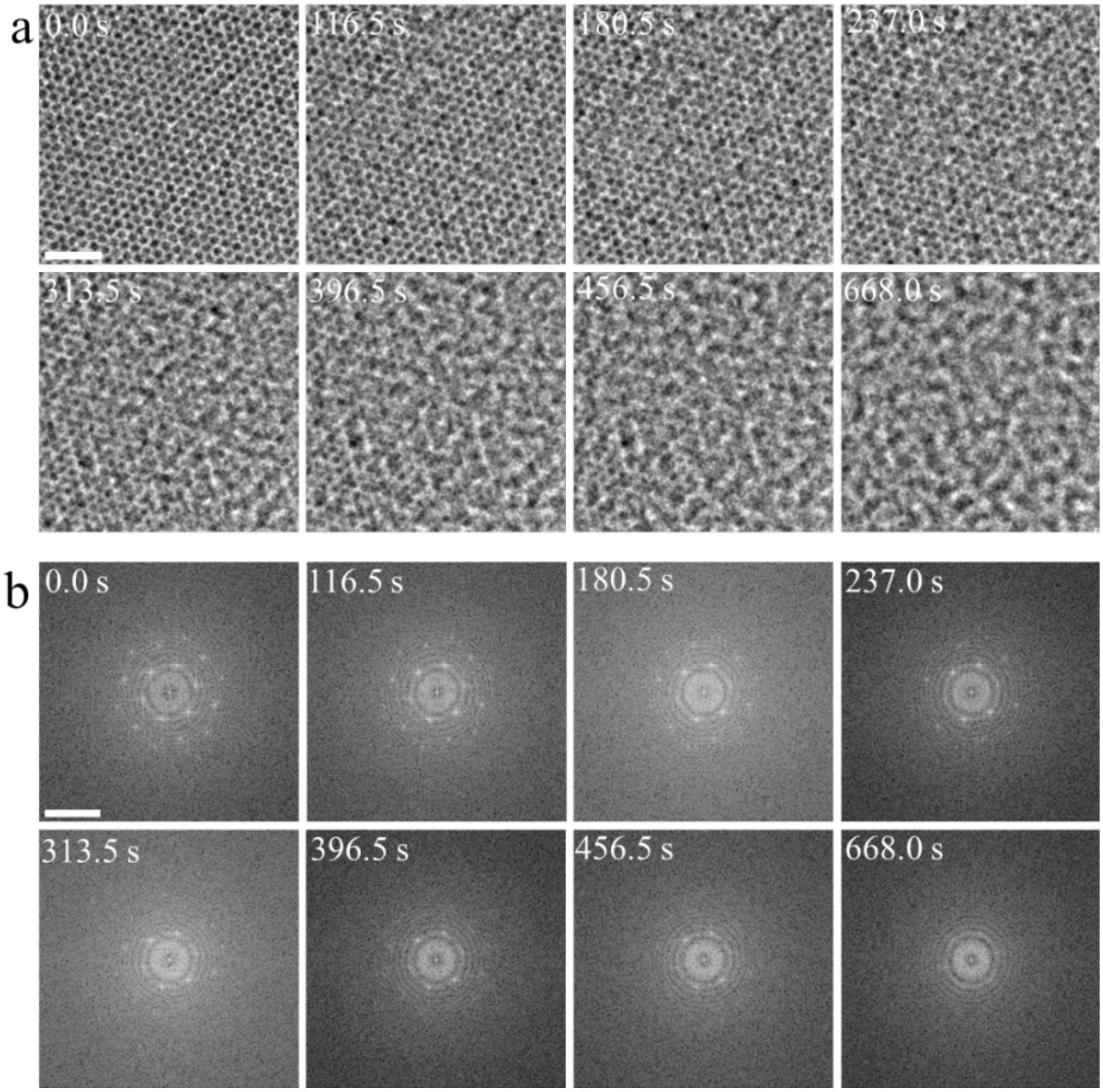

Supplementary Fig. 14 Stability test of Hf-MOLs under electron-beam irradiation at room temperature in TEM mode. a Sequential TEM images and **b** corresponding FFT images showing MOL under electron beam irradiation at a dose rate of 1750 $e^{-}\cdot Å^{-2}\cdot s^{-1}$ for over 10 mins. Scale bars: a, 10 nm; b, 1 1/nm.

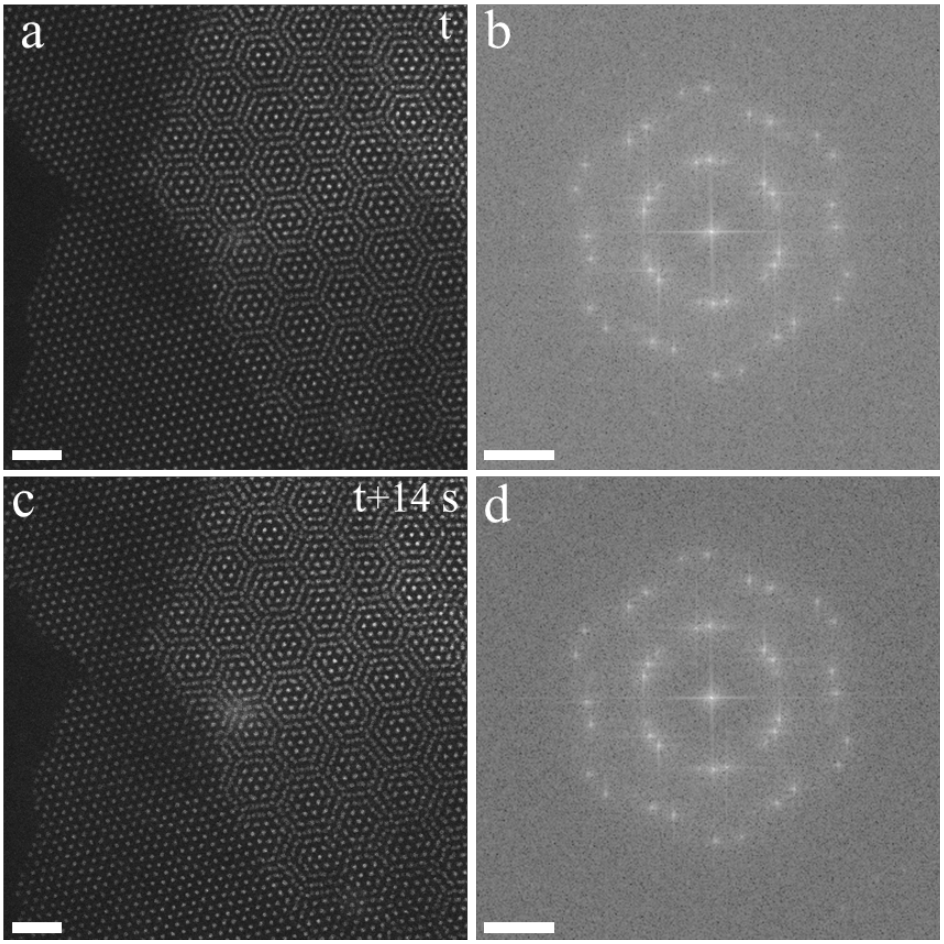

Supplementary Fig. 15 Stability test of Hf-MOLs under electron-beam irradiation at room temperature in STEM mode. a HAADF-STEM image of MOLs acquired at a dose of 106 $e^{-}\cdot Å^{-2}$ **b** FFT of the whole STEM image in a. **c** HAADF-STEM image of MOLs acquired at a dose of 106 $e^{-}\cdot Å^{-2}$ after exposed to the electron beam for 14 s at the same dose rate. **d** FFT of the whole STEM image in c. Scale bars: a, c 10 nm; b, d 0.5 1/nm.

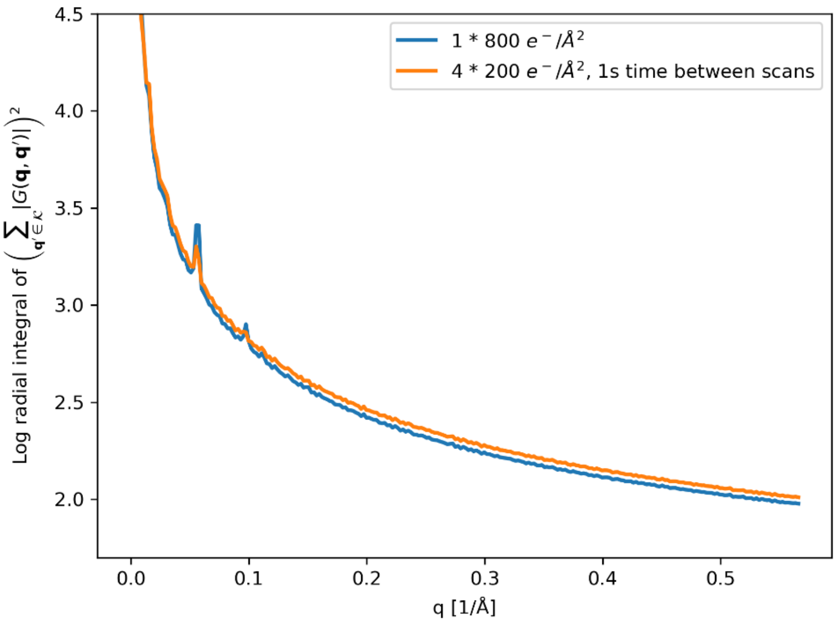

Supplementary Fig. 16 A single high-dose-rate exposure produces higher signal than many low-dose-rate exposures. During the optimization of the imaging conditions for low-dose ptychography, we found that overall higher resolution signal is present if the data was recorded in a high dose-rate regime. To corroborate this finding, we collected 4D-STEM data with a single exposure of 800 $e^{-}\cdot Å^{-2}$ with four consecutive exposures of 200 $e^{-}\cdot Å^{-2}$ of adjacent regions, such that the total dose was the same, but the dose rate was different. We observed that for the high dose-rate, the diffraction signal reached an overall higher resolution.

4D-STEM datasets recorded with Nyquist-sampled scan grid allow to analyze the maximum achievable diffraction signal by analyzing the G-function without performing a full phase retrieval. The G-function is an intermediate 4D function in the single-sideband reconstruction and defined as

$$G(\mathbf{k},\mathbf{K}) = |A(\mathbf{k})|^2\,\delta(\mathbf{k}) + A(\mathbf{k})A^*(\mathbf{k}+\mathbf{K})T(-\mathbf{k})^* - A^*(\mathbf{k})A(\mathbf{k}-\mathbf{K})T(\mathbf{k})$$

It can be calculated from the 4D-STEM data by a 2D Fourier transform along the scan coordinate axes. Using the double-overlap region

$$\mathcal{K} = \{\mathbf{q} \,:\, (|\mathbf{q}| < k_0) \wedge (|\mathbf{q}+\mathbf{q}| > k_0) \wedge (|\mathbf{q}-\mathbf{q}| < k_0)\}$$

we can calculate maximum possible diffraction signal of a certain spatial frequency as

$$\left(\sum_{\mathbf{q}' \in \mathcal{K}} |G(\mathbf{q}, \mathbf{q}')| \right)^2$$

This represents the maximum intensity in a certain spatial frequency q that can be achieved if all pixels on the detector are in phase, i.e., if the aberrations were solved perfectly.

We plot the logarithm of the radial integral of this signal below to demonstrate that diffraction information is preserved better for the Hf-MOL sample in a high-dose regime.
