# Supplementary material for: Observation of formation and local structures of metal-organic layers via complementary electron microscopy techniques
Source: Nat Commun. 2022 Sep 3;13:5197. doi: 10.1038/s41467-022-32330-z (PMC9440887; doi:10.1038/s41467-022-32330-z)
Supplement: Supplementary file 3 — Description of Additional Supplementary Files [file 41467_2022_32330_MOESM3_ESM.pdf]

Supplementary Movie 1: In-situ liquid phase TEM movie showing the cluster formation in solution (dose rate: 39.5 e-·Å-2·s-1).

Supplementary Movie 2: In-situ liquid phase TEM movie showing the slow movement of clusters (dose rate: 73.3 e-·Å-2·s-1).

Supplementary Movie 3: In-situ liquid phase TEM movie showing the self-assembly process of clusters (dose rate: 130 e-·Å-2·s-1).

Supplementary Movie 4: Stability test of Hf-MOLs under electron-beam irradiation at room temperature in TEM mode (dose rate: 1750 e-·Å-2·s-1).
